# Supplementary material for: Production of Value-Added Arabinofuranosyl Nucleotide Analogues from Nucleoside by an In Vitro Enzymatic Synthetic Biosystem
Source: Biomolecules. 2024 Nov 13;14(11):1440. doi: 10.3390/biom14111440 (PMC11591822; doi:10.3390/biom14111440)
Supplement: Supplementary file 1 [file biomolecules-14-01440-s001.zip › biomolecules-3280183-supplementary.pdf]

Supporting Information

Production of Value-Added Arabinofuranosyl Nucleotide Analogues from Nucleoside by an In Vitro Enzymatic Synthetic Biosystem

Yuxue Liu, Xiaojing Zhang, Erchu Yang, Xiaobei Liu, Weiwei Su, Zhenyu Wang *
and Hailei Wang *

Henan Engineering Research Center of Bioconversion Technology of Functional Microbes,

College of Life Science, Henan Normal University, Xinxiang 453007, China

* Correspondence: wangzhenyu@htu.edu.cn (Z.W.); whl@htu.cn (H.W.)

Table S1. The plasmids and strains used in this study**.**

Strain or plasmid	Characteristic	source
Strain		
E. coli DH5α	F^- φ80*lacZ*ΔM15 Δ(*lacZYA-argF*) U169 *deoR recA1 endA1 hsdR17*(rK^-, mK^+) *phoA supE44* λ^- *thi*$^{-1}$ *gyrA96 relA1*	Lab collection
E. coli BL21 (DE3)	F^- *ompT hsdSB* (rB^- mB^-) *gal* (λ *c I 857 ind1 sam7 nin5 lacUV5*-T7*gene1*) *dcm* (DE3)	Lab collection
Plasmid		
pET28a	Vector for protein expression; Kan^r	Lab collection
pET28a-*Ec*API	pET28a with *kdsD* gene from *E. coli*	Lab collection
pET28a-*Bc*PPM	pET28a with codon optimized *deoB* gene from *Bacillus cereus*	Lab collection
pET28a-*Kl*PNP	pET28a with *deoD* gene from *Klebsiella*	Lab collection
pET28a-*Ec*PNP	pET28a with *deoD* gene from *E. coli*	Lab collection
pET28a-*Ec*UP	pET28a with *udp* gene from *E. coli*	Lab collection
pET28a-*Aa*PNP	pET28a with *deoD* gene from *Alicyclobacillus acidoterrestris*	Lab collection
pET28a-*Hs*RK	For the expression of ribokinase isoform 1 from *Homo sapiens*	Lab collection

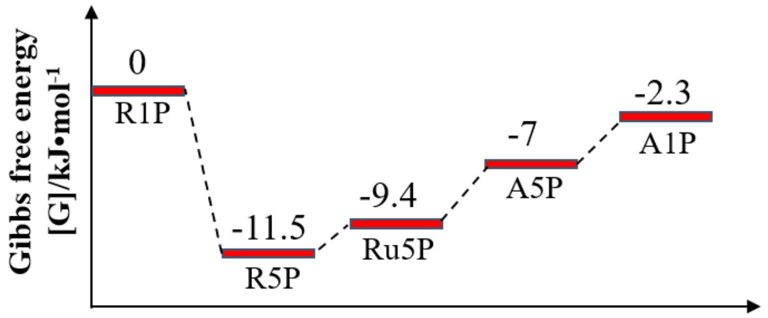

Figure S1. The changes in Gibbs free energy (ΔG°) for each individual reaction of converting R1P to A1P in this multistep biocatalytic cascade. The ΔG° was analyzed under conditions characterized by a pH of 7.5 and an ionic strength of 0.25 M (https://equilibrator.weizmann.ac.il/). Standard concentrations for reactants and products were assumed to be 1 M.

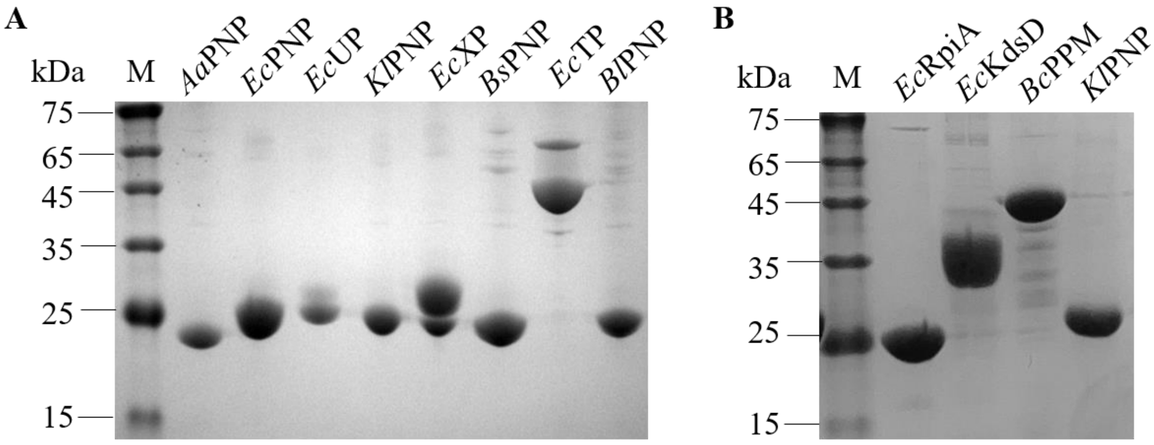

Figure S2. SDS-PAGE results of the purification of enzymes. (**A**) NPs sourced from different microorganisms (**B**) Enzymes used in the multi-enzyme system.
