# Supplementary figures and images for: Production of Value-Added Arabinofuranosyl Nucleotide Analogues from Nucleoside by an In Vitro Enzymatic Synthetic Biosystem
Source: Biomolecules. 2024 Nov 13;14(11):1440. doi: 10.3390/biom14111440 (PMC11591822; doi:10.3390/biom14111440)

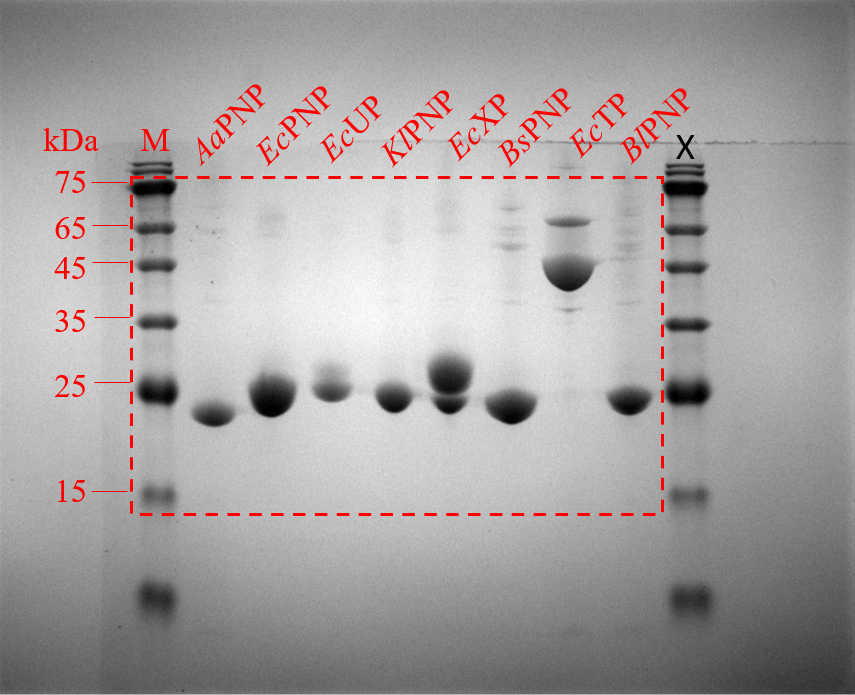

Supplement: Supplementary file 1 [file biomolecules-14-01440-s001.zip › biomolecules-3280183-original images/Figure S2A-OI.png]

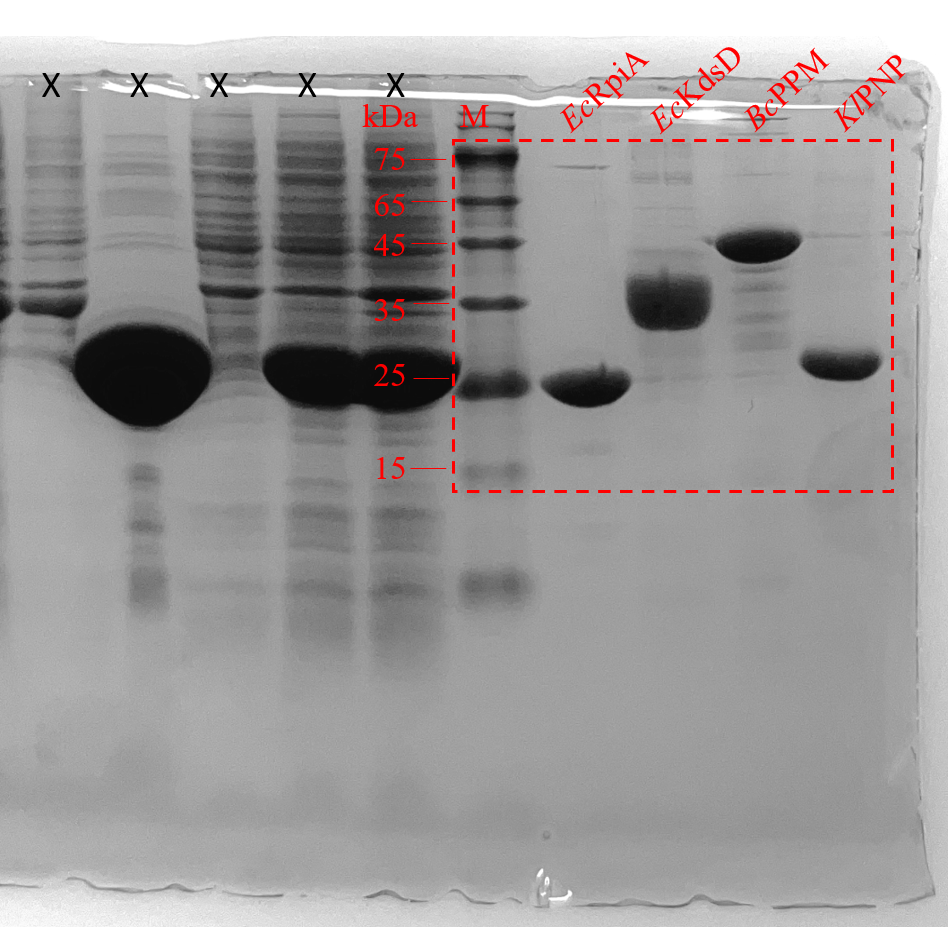

Supplement: Supplementary file 1 [file biomolecules-14-01440-s001.zip › biomolecules-3280183-original images/Figure S2B-OI.png]
